# Supplementary material for: Long-term health impacts of COVID-19 among 242,712 adults in England
Source: Nat Commun. 2023 Oct 24;14:6588. doi: 10.1038/s41467-023-41879-2 (PMC10598213; doi:10.1038/s41467-023-41879-2)
Supplement: Supplementary file 1 — Supplementary Information [file 41467_2023_41879_MOESM1_ESM.pdf]

Long-term health impacts of COVID-19 among 242,712 adults in England

Supplementary material

Supplementary Figure 1: Study population flowchart 2

Supplementary Table 1: Sample frame and response of participants 3

Supplementary Table 2: Factors associated with persistent COVID-19 symptoms lasting i) ≥12 weeks and ii) ≥52 weeks 4

Supplementary Table 3: Association between dominant strain at symptom onset (or positive test) and persistent COVID-19 symptoms lasting ≥12 weeks 7

Supplementary Table 4: Survival analysis with Accelerated Time Failure model: Factors associated with likelihood (adjusted Time Ratio) of longer duration of symptoms in study participants with symptomatic SARS-CoV-2 infection lasting ≥12 weeks (N=8,532) 9

Supplementary Table 5: Modelling of current symptoms in those reporting ongoing persistent symptoms following COVID-19 versus all other respondents (n=242,712) 11

Supplementary Table 6: Prevalence of current symptoms in the study population by COVID-19 history (n=242,712) 12

Supplementary Table 7: Association between COVID-19 history and current symptom profile and health-related quality of life characteristics adjusted for age, sex, ethnicity, IMD, comorbidities, smoking status. Odds Ratios with 95% CI (White Cells) and Regression Coefficients, 95% CI (Grey Cells) 14

Supplementary Table 8: Current PHQ-2 of participants by COVID-19 history (n=242,712) 16

References 16

Supplementary Figure 1: Study population flowchart

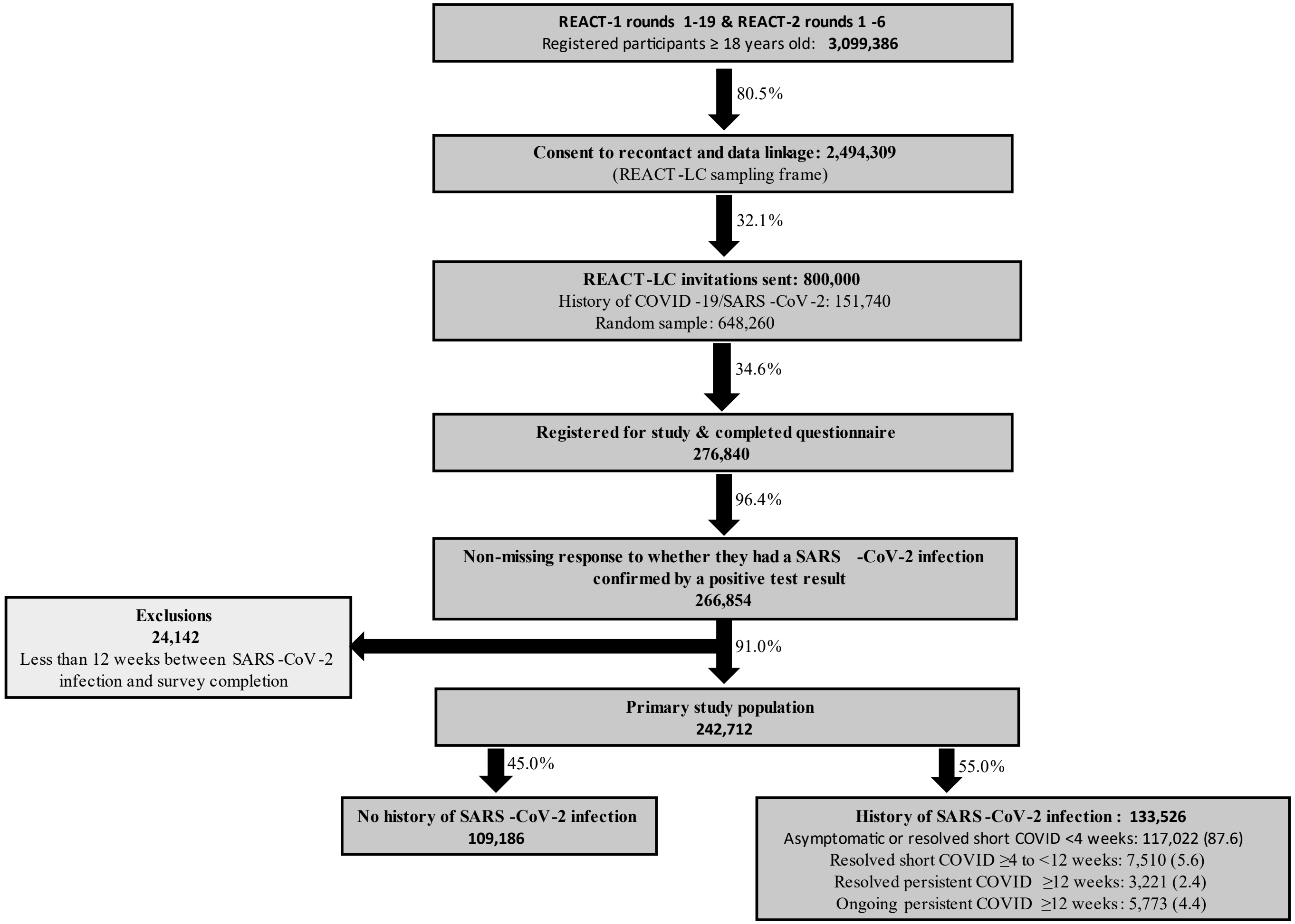

Supplementary Table 1: Sample frame and response of participants

	**England (18+ population)[1, 2]**	**REACT-1/-2 (18+ population)**	**Target population**	**REACT-LC participants**
	N= 43,752,473	N=3,099,386	N=800,000	N=276,840
**Sex**				
Male	48.9	44.9	43.2	41.3
Female	51.1	55.1	56.8	58.7
**Age**				
18-24	10.9	5.7	5.0	2.8
25-34	17.3	12.2	10.7	6.7
35-44	16.1	15.3	15.1	11.5
45-54	17.5	18.4	18.8	17.4
55-64	15.0	21.4	22.4	25.9
65-74	12.6	18.4	18.8	24.8
75+	10.5	8.5	9.3	10.9
**Ethnicity**				
White	86.3	91.8	93.5	94.6
Mixed	1.1	1.3	1.3	1.2
Asian	7.3	4.5	3.5	2.7
Black	3.3	1.4	0.9	0.8
Other	1.9	0.93	0.8	0.7
**Region**				
North East	4.8	3.8	3.9	3.8
North West	13.0	11.9	11.7	11.0
Yorkshire and The Humber	9.8	6.7	7.4	7.5
East Midlands	8.7	12.7	11.5	11.2
West Midlands	10.5	9.4	9.4	9.1
East of England	11.1	14.4	13.7	13.7
London	15.6	9.9	11.0	10.9
South East	16.3	21.7	21.3	21.9
South West	10.2	9.6	10.1	10.8
**IMD Quintile**				
1 – most deprived	19.8	11.2	9.8	8.4
2	20.9	16.8	15.8	15.1
3	20.4	21.5	21.5	21.4
4	19.7	24.2	24.9	25.4
5 – least deprived	19.1	26.4	28.0	29.8

Supplementary Table 2: Factors associated with persistent COVID-19 symptoms lasting i) ≥12 weeks and ii) ≥52 weeks

	Controls: asymptomatic or resolved short COVID <4 weeks	Cases 1: persistent COVID ≥12 weeks	Unadjusted OR (95% CI)	P-value	$Adjusted OR (95% CI)	P-value	Cases 2: persistent COVID ≥52 weeks	Unadjusted OR (95% CI)	P-value	$Adjusted OR (95% CI)	P-value
	N=117,022	N=8,994					N= 4,120				
**Age**											
18 to 24	3,887	350	REF		REF		176	REF		REF	
	3.32	3.89					4.27				
25 to 34	9,831	808	0.91 (0.80, 1.04)	0.17	0.95 (0.82, 1.11)	0.55	334	0.75 (0.62, 0.90)**	0.0003	0.86 (0.69, 1.07)	0.18
	8.40	8.98					8.11				
35 to 44	16,971	1,649	1.08 (0.96, 1.22)	0.22	1.12 (0.97, 1.28)	0.13	711	0.93 (0.78, 1.10)	0.37	1.10 (0.90, 1.34)	0.36
	14.50	18.33					17.26				
45 to 54	23,319	2,230	1.06 (0.94, 1.19)	0.32	1.00 (0.88, 1.15)	0.95	1,028	0..97 (0.83, 1.15)	0.75	1.02 (0.84, 1.23)	0.88
	19.93	24.79					24.95				
55 to 64	30,139	2,355	0.87 (0 .77, 0.98)*	0.02	0.87 (0.75, 0.99)*	0.04	1,134	0.83 (0.71, 0.98)*	0.03	0.92 (0.75, 1.11)	0.37
	25.75	26.18					27.52				
65 to 74	24,828	1,230	0.55 (0.49, 0.62)***	0.000002	0.74 (0.64, 0.86)***	0.00005	577	0.51 (0.43, 0.61)***	0.0000003	0.84 (0.69, 1.03)	0.10
	21.22	13.68					14.00				
75+	8,047	372	0.51 (0.44, 0.60)***	0.0000005	0.84 (0.70, 0.99)*	0.04	160	0.44 (0.35, 0.55)***	0.0000001	0.86 (0.66, 1.11)	0.24
	6.88	4.14					3.88				
**Sex at birth**											
Male	47,368	2,749	REF		REF		1,287	REF		REF	
	40.48	30.56					31.24				
Female	69,651	6,245	1.54 (1.47, 1.62)***	0.000002	1.42 (1.35, 1.50)***	0.000002	2,833	1.50 (1.40, 1.60)***	0.0000003	1.49 (1.38, 1.62)***	0.0000003
	59.52	69.44					68.76				
**Ethnicity**											
White	109,600	8,352	REF		REF		3,828	REF		REF	
	94.59	93.77					94.05				
Mixed	1,507	143	1.25 (1.04, 1.48)*	0.01	0.99 (0.81, 1.21)	0.93	51	0.97 (0.73, 1.28)	0.83	0.71 (0.51, 1.00)	0.06
	1.30	1.61					1.25				
Asian	3,085	250	1.06 (0.93, 1.21)	0.36	0.80 (0.69 , 0.93)**	0.0004	115	1.07 (0.88, 1.29)	0.50	0.71 (0.57, 0.88)**	0.0002
	2.66	2.81					2.83				
Black	846	77	1.19 (0.94, 1.51)	0.14	0.93 (0.71, 1.21)	0.58	41	1.39 (1.01, 1.90)*	0.04	0.95 (0.66, 1.37)	0.80
	0.73	0.86					1.01				
Other	833	85	1.34 (1.07, 1.68)*	0.01	0.91 (0.70, 1.19)	0.50	35	1.20 (0.86, 1.69)	0.29	0.72 (0.48, 1.06)	0.10
	0.72	0.95					0.86				
**IMD**											
1: most deprived	9,473	1,067	REF		REF		564	REF		REF	
	8.31	12.11					13.99				
2	17,063	1,508	0.78 (0.72, 0.85)***	0.000008	0.90 (0.82, 0.99)*	0.04	710	0.70 (0.62, 0.78)***	0.0000006	0.87 (0.76, 0.99)*	0.04
	14.97	17.12					17.61				

3	24,032	1,865	0.69 (0.64, 0.75)***	0.000002	0.87 (0.79, 0.95)**	0.0002	856	0.60 (0.54, 0.67)***	0.0000002	0.83 (0.73, 0.95)**	0.0005
	21.09	21.17					21.24				
4	29,103	2,081	0.63 (0.59, 0.69)***	0.000001	0.83 (0 76, 0.91)***	0.00004	933	0.54 (0.48, 0.60)***	0.0000009	0.78 (0.69, 0.89)***	0.00001
	25.54	23.62					23.15				
5: least deprived	34,294	2,288	0.59 (0.55, 0.64)***	0.000002	0 82 (0.75, 0.90)***	0.00001	968	0.47 (0.43, 0.53)***	0.0000004	0.75 (0.66 , 0.85)***	0.000007
	30.09	25.97					24.01				
**Comorbidities**											
0	106,790	8,130	REF		REF		3,723	REF		REF	
	91.26	90.39					90.36				
1	7,402	637	1.13 (1.04, 1.23)**	0.0004	1.31 (1.19, 1.44)***	0.000004	274	1.06 (0.94, 1.20)	0.35	1.52 (1.31, 1.76)***	0.0000003
	6.33	7.08					6.65				
2 or more	2,830	227	1.05 (0.92, 1.21)	0.46	1.46 (1.27, 1.75)***	0.000001	123	1.25 (1.04, 1.50)*	0.02	2.35 (1.85, 2.97)***	0.0000002
	2.42	2.52					2.99				
**Smoking Status**											
Current smoker	5,365	463	REF		REF		213	REF		REF	
	4.86	5.56					5.62				
Not current smoker	105,037	7,860	0.87 (0.79, 0.96)**	0.0004	0.98 (0.88, 1.09)	0.74	3,580	0.86 (0.75, 0.99)*	0.03	0.99 (0.84, 1.15)	0.86
	95.14	94.44					94.38				
**Severity of initial SARS-CoV-2 infection**											
No symptoms	6,206	0	omitted		omitted		0	omitted		omitted	
	5.32	0.0					0.00				
Mild symptoms	34,346	1,137	REF		REF		564	REF		REF	
	29.47	12.64					13.69				
Moderate symptoms	58,023	3,627	1.89 (1.76, 2.02)***	0.000004	1.76 (1.63, 1.89)***	0.000001	1,537	1.61 (1.46, 1.78)***	0.0000007	1.47 (1.32, 1.64)***	0.0000006
	49.78	40.33					37.31				
Severe symptoms	17,974	4,230	7.11 (6.64, 7.61)***	0.000001	4.87 (4.52, 5.25)***	0.000001	2,019	6.84 (6.22, 7.52)***	0.0000001	3.55 (3.19, 3.96)***	0.0000004
	15.42	47.03					49.00				
**Dominant strain at symptom onset or positive test**											
Wild type (before Dec 2020)	10,157	3,093	REF		REF		2,185	REF		REF	
	8.89	34.39					53.03				
Alpha (Dec 2020-April 2021)	9,124	1,666	0.60 (0.56, 0.64)***	0.000003	0.60 (0.56, 0.64)***	0.000002	1,158	0.59 (0.55, 0.64)***	0.0000002	0.59 (0.54, 0.64)***	0.0000006
	7.99	18.52					28.11				
Delta (May 2021-mid-Dec 2021)	16,679	1,850	0.36 (0.34, 0.39)***	0.000005	0.38 (0.35, 0.41)***	0.000002	777	0.22 (0.20, 0.24)***	0.0000002	0.32 (0.29, 0.36)***	0.0000001

	14.60	20.57					18.86				
Omicron (after mid-Dec 2021)	78,301	2,385	0.10 (0.09, 0.11)***	0.000001	0.12 (0.11, 0.13)***	0.000001	0	Omitted		Omitted	
	68.53	26.52					0.00				
**Vaccination status at symptom onset or positive test**											
0	56,079	6,281	REF		REF		3,678	REF		REF	
	47.92	69.84					89.27				
1	1,804	218	1.08 (0.94, 1.24)	0.30	0.98 (0.84, 1.15)	0.84	150	1.27 (1.07, 1.50)**	0.0006	1.06 (0.88, 1.29)	0.52
	1.54	2.42					3.64				
2 or more	59,139	2,495	0.38 (0.36, 0.40)***	0.000001	0.94 (0.87, 1.00)	0.06	292	0.08 (0.07, 0.09)***	0.0000001	0.43 (0.37, 0.51)***	0.0000008
	50.54	27.74					7.09				

Logistic regression models with one or more COVID-19 symptoms lasting ≥12 weeks (y/n) (n=126,016 participants) or ≥52 weeks (y/n) (n=121,142 participants) as the binary outcome variables. Modelling of persistent symptoms as a function of biological and demographic variables. Odds ratio compares participants with persistent symptoms lasting i) ≥12 weeks or ii) ≥52 weeks with those who reported being asymptomatic or symptoms resolved within 4 weeks. [$]Mutually adjusted for age, sex, ethnicity, IMD, comorbidities, smoking status, severity of initial infection, dominant variant at time of infection, vaccination status. No adjustments were made for multiple comparisons. *p<0·05, **p<0·01, ***p<0·0001.

Supplementary Table 3: Association between dominant strain at symptom onset (or positive test) and persistent COVID-19 symptoms lasting ≥12 weeks

We cannot rule out residual confounding from other unmeasured time-varying factors, such as behaviour, seasonal weather patterns and changing pandemic restrictions, knowledge and expectations, for the association between persistent symptoms and dominant variant circulating at the time of infection Thus, further analysis restricting the comparison to participants infected close to transition periods between variants was performed. The Wild-type strain was dominant in the UK prior to 1st December 2020. Alpha dominated between 1st December 2020 and 30 April 2021 followed by Delta (1st May 2021 to 15th December 2021 and Omicron (16th December 2021 onwards).[3-5] In this analysis we only included participants with a confirmed COVID-19 infection date within the following "one month" time periods, excluding a two week interval either side of the transition dates used:

Wild-type: Oct 16th 2020 – Nov 15th 2020

Alpha-1: Dec 16th 2020– Jan 15th 2021

Alpha-2: Mar 16th 2021– April 15th 2021

Delta-1: May 16th 2021– June 15th 2021

Delta-2: Nov 16th 2021- Nov 30th 2021

Omicron: Jan 1st 2022 – Jan 15th 2022

The Odds Ratios (OR) in the table compare participants with persistent symptoms lasting ≥12 weeks with those who reported being asymptomatic or symptoms resolved within 4 weeks. To limit residual confounding from other unmeasured time-varying factors, the following dominant variant time periods were compared:

1. Wild-type: Oct 16th 2020 – Nov 15th 2020 verses Alpha-1: Dec 16th 2020– Jan 15th 2021
2. Alpha-2: Mar 16th 2021– April 15th 2021 verses Delta-1: May 16th 2021– June 15th 2021
3. Delta-2: Nov 16th 2021- Nov 30th 2021 verses Omicron: Jan 1st 2022 – Jan 15th 2022

	**Controls: asymptomatic or resolved short COVID <4 weeks**	**Cases: persistent COVID ≥12 weeks**	**Unadjusted OR (95% CI)**	***P* value**	**[$]Adjusted OR (95% CI)**	***P* value**
	N=19,844	N=2,233				
**Dominant strain at symptom onset or positive test**						
Wild type	1,882	561	REF		REF	
	77.0	23.0				

Alpha - 1	4,047	794	0.66 (0.58, 0.74)***	0.00000001	0.63 (0.55, 0.72)***	0.0000009
	83.6	16.4				
Alpha -2	1,107	132	REF		REF	
	89.4	10.6				
Delta - 1	796	105	1.11 (0.84, 1.45)	0.47	0.86 (0.61, 1.21)	0.38
	88.4	11.6				
Delta-2	3,226	325	REF		REF	
	90.9	9.1				
Omicron	8,786	316	0.36 (0.30, 0.42)***	0.00000002	0.44 (0.37, 0.52)***	0.00000004
	96.5	3.5				

Logistic regression models with one or more COVID-19 symptoms lasting ≥12 weeks (y/n) as the binary outcome variable. Modelling of persistent symptoms as a function of dominant strain circulating in the UK at symptom onset. $Mutually adjusted for age, sex, ethnicity, IMD, comorbidities, smoking status, severity of initial infection, dominant variant at time of infection, vaccination status. No adjustments were made for multiple comparisons. *p<0·05, **p<0·01, ***p<0·0001.

Supplementary Table 4: Survival analysis with Accelerated Time Failure model: Factors associated with likelihood (adjusted Time Ratio) of longer duration of symptoms in study participants with symptomatic SARS-CoV-2 infection lasting ≥12 weeks (N=8,532)

	N	Unadjusted TR (95% CI)	*P* value	$Adjusted TR (95% CI)	*P* value
**Age**					
18 to 24	329	REF		REF	
25 to 34	757	1.04 (0.85, 1.28)	0.68	0.95 (0.77, 1.18)	0.66
35 to 44	1,551	1.04 (0.86, 1.26)	0.67	0.99 (0.81, 1.21)	0.91
45 to 54	2,123	1.01 (0.84, 1.22)	0.88	0.95 (0.78, 1.15)	0.60
55 to 64	2,225	0.95 (0.79, 1.14)	0.59	0.91 (0.75, 1.11)	0.35
65 to 74	1,191	0.91 (0.75, 1.10)	0.33	0.91 (0.74, 1.11)	0.34
75+	356	0.97 (0.76, 1.23)	0.80	0.98 (0.76, 1.26)	0.86
**Sex at birth**					
Male	2,613	REF		REF	
Female	5,919	1.13 (1.05, 1.21)**	0.001	1.14 (1.06, 1.23)**	0.00006
**Ethnicity**					
White	7,917	REF		REF	
Mixed	136	0.80 (0.62, 1.04)	0.10	0.75 (0.57, 0.99)*	0.04
Asian	242	0.87 (0.72, 1.06)	0.18	0.85 (0.69, 1.05)	0.13
Black	75	0.90 (0.64, 1.27)	0.55	0.80 (0.56, 1.14)	0.22
Other	78	0.69 (0.50, 0.96)*	0.03	0.63 (0.45, 0.89)**	0.008
**IMD**					
1 – most deprived	1,007	REF		REF	
2	1,424	0.86 (0.76, 0.98)*	0.03	0.91 (0.80, 1.04)	0.16
3	1,768	0.79 (0.70, 0.89)***	0.00002	0.81 (0.71, 0.92)**	0.0001
4	1,962	0.82 (0.72, 0.93)**	0.0001	0.89 (0.78, 1.01)	0.07
5 – least deprived	2,197	0.72 (0.64, 0.81)***	0.0000001	0.78 (0.69, 0.88)**	0.009
**Comorbidities**					
0	7,704	REF		REF	
1	612	1.21 (1.06, 1.39)**	0.006	1.24 (1.08, 1.42)**	0.0003
2 or more	216	2.11 (1.64, 2.71)***	0.00000007	2.05 (1.58, 2.66)***	0.0000006
**Smoking Status**					
Current smoker	441	REF		REF	
Not current smoker	7,452	0.70 (0.59, 0.83)***	0.00005	0.73 (0.62, 0.86)***	0.00001

**Severity of initial SARS-CoV-2 infection**					
No symptoms	0	omitted		omitted	
Mild symptoms	1,016	REF		REF	
Moderate symptoms	3,410	0.93 (0.84, 1.04)	0.22	0.90 (0.81, 1.01)	0.08
Severe symptoms	4,106	1.05 (0.94, 1.17)	0.37	1.00 (0.90, 1.12)	0.96
**Dominant strain at symptom onset or positive test**					
Wild type (before Dec 2020)	3,074	REF		REF	
Alpha (Dec 2020-April 2021)	1,635	0.82 (0.75, 0.90)***	0.00001	0.79 (0.72, 0.86)***	0.0000003
Delta (May 2021-mid-Dec 2021)	1,786	0.89 (0.81, 0.97)*	0.01	0.89 (0.79, 0.99)*	0.04
Omicron (after mid-Dec 2021)	2,037	0.70 (0.63, 0.76)***	0.00000002	0.69 (0.61, 0.78)***	0.000000002
**Vaccination status at symptom onset or positive test**					
0	6,068	REF		REF	
1	208	1.00 (0.80, 1.23)	0.97	1.10 (0.87, 1.38)	0.43
2 or more	2,256	0.86 (0.79, 0.93)***	0.00002	0.98 (0.88, 1.10)	0.77

Accelerated Failure Time model with a Log-Normal distribution used to quantify the associations between COVID-19 symptom discontinuation beyond 12 weeks and biological and demographic variables. An aTR >1 is interpreted as a slower symptom recovery rate beyond 12 weeks in participants with COVID-19 symptoms lasting ≥12 weeks. $^{\$}$ Mutually adjusted for age, sex, ethnicity, IMD, comorbidities, smoking status, severity of initial infection, dominant variant at time of infection, vaccination status. No adjustments were made for multiple comparisons. *p<0·05, **p<0·01, ***p<0·0001.

Supplementary Table 5: Modelling of current symptoms in those reporting ongoing persistent symptoms following COVID-19 versus all other respondents (n=242,712)

**Symptom**	**Adjusted Odds Ratio (95% CI)**	***P*-value**
Skin issues (itchy, scaly, redness, etc)	2.27 (2.13, 2.42)	0.000003
Hearing issues (e.g. hearing loss, Tinnitus etc)	2.27 (2.14, 2.42)	0.00000001
Fever	2.33 (2.06, 2.63)	0.000005
Itchy, sore or red eyes, conjunctivitis	2.34 (2.19, 2.49)	0.00009
Headaches	2.46 (2.32, 2.60)	0.000007
Difficulty sleeping	2.47 (2.34, 2.62)	0.00002
Leg swelling (Thrombosis)	2.52 (2.25, 2.82)	0.0000004
Achy or cramping muscles, pain in muscles	2.56 (2.42, 2.70)	0.00000003
Low mood	2.62 (2.47, 2.79)	0.0000005
Anxiety	2.71 (2.55, 2.88)	0.0000003
Pain in joints	2.71 (2.56, 2.87)	0.000006
Hair loss	2.75 (2.50, 3.01)	0.0000008
Coughing	2.82 (2.62, 3.03)	0.00000004
Mood swing	2.84 (2.65, 3.03)	0.00007
Red/purple sores or blisters on your feet (including toes)	2.85 (2.31, 3.52)	0.0000002
Numbness or tingling somewhere in the body	3.09 (2.90, 3.30)	0.0000004
Appetite loss	3.30 (3.05, 3.57)	0.000009
Vision issues	3.40 (3.15, 3.67)	0.00001
Sudden swelling of the face or lips	3.44 (2.62, 4.52)	0.00006
Dizziness, vertigo	3.46 (3.24, 3.69)	0.000006
Heart issues (racing heart, palpitations, irregular heartbeat etc)	3.48 (3.26, 3.71)	0.00000009
Mild fatigue (e.g. feeling tired)	3.66 (3.45, 3.89)	0.0000005
Poor memory	4.40 (4.15, 4.66)	0.00007
Tightness or heaviness in chest, chest pain	4.71 (4.37, 5.08)	0.0000008
Difficulty thinking or concentrating	4.97 (4.68, 5.27)	0.000005
Severe fatigue (e.g. inability to get out of bed)	6.19 (5.66, 6.77)	0.0000003
Shortness of breath, breathlessness, wheezing	6.69 (6.29, 7.12)	0.00000001
Loss or change of sense of taste	8.47 (7.85, 9.15)	0.000000002
Loss or change of sense of smell	9.31 (8.64, 10.04)	0.000000004

Logistic regression models with 29 individual symptoms currently experienced (y/n) as the binary outcome variable and COVID-19 history as the primary exposure variable of interest. Odds ratio compares participants with ongoing persistent symptoms lasting ≥12 weeks with all other participants. $Mutually adjusted for age, sex, ethnicity, IMD, comorbidities, smoking status, severity of initial infection, dominant variant at time of infection, vaccination status. No adjustments were made for multiple comparisons.

Supplementary Table 6: Prevalence of current symptoms in the study population by COVID-19 history (n=242,712)

Symptom	No COVID n (%)	Asymptomatic or resolved short COVID <4 n (%)	Resolved short COVID ≥4 to <12 n (%)	Resolved persistent COVID ≥12 n (%)	Ongoing persistent COVID ≥12 n (%)
	109,186	117,022	7,510	3,221	5,773
Skin issues (itchy, scaly, redness, etc)	15,684 (14.4)	16,963 (14.5)	1,382 (18.4)	600 (18.6)	1,488 (25.8)
Hearing issues (e.g. hearing loss, Tinnitus etc)	17,960 (16.5)	18,535 (15.8)	1,464 (19.5)	628 (19.5)	1,551 (26.9)
Fever	1,925 (1.8)	3,045 (2.6)	288 (3.8)	142 (4.4)	352 (6.1)
Itchy, sore or red eyes, conjunctivitis	14,961 (13.7)	16,029 (13.7)	1,328 (17.7)	572 (17.8)	1,460 (25.3)
Headaches	23,991 (22.0)	32,483 (27.8)	2,653 (35.3)	1,109 (34.4)	2,829 (49.0)
Difficulty sleeping	29,106 (26.7)	34,746 (29.7)	2,897 (38.6)	1,271 (39.5)	2,875 (49.8)
Leg swelling (Thrombosis)	4,700 (4.3)	3,913 (3.3)	380 (5.1)	159 (4.9)	403 (7.0)
Achy or cramping muscles, pain in muscles	30,055 (27.5)	33,003 (28.2)	2,659 (35.4)	1,068 (33.2)	2,783 (48.2)
Low mood	17,678 (16.2)	22,713 (19.4)	2,010 (26.8)	824 (25.6)	2,186 (37.9)
Anxiety	19,360 (17.7)	24,435 (20.9)	2,182 (29.1)	928 (28.8)	2,298 (39.8)
Pain in joints	38,762 (35.5)	40,222 (34.4)	3,177 (42.3)	1,312 (40.7)	3,154 (54.6)
Hair loss	3,717 (3.4)	4,930 (4.2)	492 (6.6)	223 (6.9)	655 (11.4)
Coughing	7,272 (6.7)	9,763 (8.3)	829 (11.0)	332 (10.3)	1,086 (18.8)
Mood swing	8,810 (8.1)	12,975 (11.1)	1,257 (16.7)	508 (15.8)	1,493 (25.9)
Red/purple sores or blisters on your feet (including toes)	748 (0.69)	808 (0.69)	84 (1.1)	37 (1.2)	106 (1.8)
Numbness or tingling somewhere in the body	12,223 (11.2)	13,036 (11.1)	1,154 (15.4)	408 (14.9)	1,477 (25.6)
Appetite loss	5,845 (5.4)	6,970 (6.0)	682 (9.1)	302 (9.4)	975 (16.9)
Vision issues	6,778 (6.2)	7,277 (6.2)	704 (9.4)	305 (9.5)	951 (16.5)
Sudden swelling of the face or lips	355 (0.33)	403 (0.34)	46 (0.61)	17 (0.53)	66 (1.1)
Dizziness, vertigo	9,768 (9.0)	11,749 (10.0)	1,113 (14.8)	506 (15.7)	1,561 (27.0)
Heart issues (racing heart, palpitations, irregular heartbeat etc)	8,482 (7.8)	10,944 (9.4)	1,035 (13.8)	455 (14.1)	1,453 (25.2)
Mild fatigue (e.g. feeling tired)	33,940 (31.1)	44,849 (38.3)	3,904 (52.0)	1,601 (49.7)	3,864 (66.9)
Poor memory	13,634 (12.5)	18,672 (16.0)	1,875 (25.0)	775 (24.1)	2,485 (43.1)
Tightness or heaviness in chest, chest pain	4,351 (4.0)	5,961 (5.1)	623 (8.3)	267 (8.3)	1,053 (18.2)
Difficulty thinking or concentrating	16,642 (15.2)	24,918 (21.3)	2,504 (33.3)	1,027 (31.9)	3,171 (54.9)
Severe fatigue (e.g. inability to get out of bed)	2,652(2.4)	2,895 (2.5)	334 (4.5)	143 (4.4)	753 (13.1)
Shortness of breath, breathlessness, wheezing	6,920 (6.3)	9,650 (8.3)	1,097 (14.6)	464 (14.4)	1,921 (33.3)

Loss or change of sense of taste	2,208 (2.0)	3,663 (3.1)	417 (5.6)	139 (4.3)	1,094 (19.0)
Loss or change of sense of smell	1,930 (1.8)	3,561 (3.0)	392 (5.2)	139 (4.3)	1,182 (20.5)

Unweighted survey data presented. Percentages are calculated by category after exclusion of missing data for that variable.

Supplementary Table 7: Association between COVID-19 history and current symptom profile and health-related quality of life characteristics adjusted for age, sex, ethnicity, IMD, comorbidities, smoking status. Odds Ratios with 95% CI (White Cells) and Regression Coefficients, 95% CI (Grey Cells)

	**No COVID**	**Asymptomatic or resolved short COVID <4 weeks (Reference Group)**	**Resolved short COVID ≥4 to <12 weeks**	**Resolved persistent COVID ≥12 weeks**	**Ongoing persistent COVID ≥12 weeks**
**Health Status**					
Good/Fair	-	-	-	-	-
Bad	0.37 (0.34, 0.39)	-	1.73 (1.53, 1.96)	1.40 (1.13, 1.72)	4.95 (4.50, 5.45)
**No. of current symptoms**	-1.72 (-1.76, -1.68)	-	1.33 (1.25, 1.42)	1.18 (1.06, 1.31)	4.12 (4.02, 4.21)
**Reduction in daily activities**					
No	-	-	-	-	-
Yes	0.57 (0.55, 0.59)	-	1.68 (1.58, 1.78)	1.48 (1.36, 1.61)	3.56 (3.29, 3.86)
[1]**Dyspnoea 12**					
Total Score	-2.37 (-2.78, -1.96)	-	1.16 (0.58, 1.74)	0.16 (-0.71, 1.03)	4.03 (3.57, 4.49)
Physical Score	-1.29 (-1.53, -1.05)	-	0.67 (0.34, 1.01)	0.23 (-0.28, 0.74)	2.20 (1.93, 2.47)
Affective Score	-1.02 (-1.20, -0.84)	-	0.47 (0.21, 0.72)	0.05 (-0.34, 0 .44)	1.82 (1.62, 2.02)
[2]**PEM Questions**					
Worsening of fatigue symptoms after minimal physical effort					
No	-	-	-	-	-
Yes	0.46 (0.44, 0.49)	-	1.54 (1.43, 1.65)	1.48 (1.33, 1.66)	3.87 (3.58, 4.18)
Worsening of fatigue symptoms after minimal mental effort					
No	-	-	-	-	-
Yes	0.53 (0.51, 0.56)	-	1.61 (1.49, 1.73)	1.57 (1.41, 1.76)	2.92 (2.70, 3.15)
Exercise makes fatigue symptoms worse					
No	-	-	-	-	-
Yes	0.53 (0.50 , 0.56)	-	1.49 (1.39, 1.61)	1.34 (1.20, 1.50)	3.44 (3.18, 3.72)

**Sleep Quality**	0.52 (0.50, 0.54)	-	-0.38 (-0.43, -0.34)	-0.46 (-0.53, -0.39)	-0.91 (-0.96, -0.86)
**EQ-5D-5L**					
EQ5D Visual Analogue	6.24 (6.05, 6.42)	-	-4.35 (-4.75, -3.94)	-3.75 (-4.37, -3.14)	-12.23 (-12.70, -11.77)
Mobility: Any problem					
No	-	-	-	-	-
Yes	0.45 (0.44, 0.47)	-	1.58 (1.49, 1.67)	1.53 (1.40, 1.67)	3.39 (3.19, 3.59)
Self-care: Any problem					
No	-	-	-	-	-
Yes	0.43 (0.41, 0.45)	-	1.61 (1.48, 1.76)	1.68 (1.47, 1.92)	3.63 (3.36, 3.93)
Usual activities: Any problem					
No	-	-	-	-	-
Yes	0.40 (0.39, 0.41)	-	1.84 (1.75, 1.94)	1.74 (1.61, 1.88)	4.86 (4.58, 5.15)
Pain / discomfort: Any problem					
No	-	-	-	-	-
Yes	0.51 (0.49, 0.52)	-	1.58 (1.50, 1.66)	1.49 (1.38, 1.61)	2.90 (2.72, 3.09)
Anxiety / depression: Any problem					
No	-	-	-	-	-
Yes	0.42 (0.41, 0.44)	-	1.64 (1.56,1.72)	1.44 (1.34, 1.56)	2.53 (2.38, 2.69)
EuroQL-5D Utility Index	0.06 (0.054, 0.057)	-	-0.04 (-0.044, -0.037)	-0.03 (-0.040, -0.029)	-0.12 (-.121, -0.113)
**PHQ-9 (>=10)**					
No	-	-	-	-	-
Yes	0.35 (0.34, 0.37)	-	1.88 (1.76, 2.00)	1.70 (1.54, 1.86)	4.11 (3.85, 4.39)
**PHQ-2 (>=3)**					
No	-	-	-	-	-
Yes	0.60 (0.58, 0.61)	-	1.67 (1.59, 1.77)	1.36 (1.25, 1.47)	2.86 (2.66, 3.07)
**GAD-7 (>=10)**					
No	-	-	-	-	-
Yes	0.36 (0.34, 0.37)	-	1.68 (1.56, 1.80)	1.56 (1.40, 1.74)	2.72 (2.53, 2.93)

Supplementary Table 8: Current PHQ-2 of participants by COVID-19 history (n=242,712)

PHQ-2 (>=3)*	No COVID<br>No. (%)	Asymptomatic or resolved short COVID <4 weeks<br>No. (%)	Resolved short COVID ≥4 to <12 weeks<br>No. (%)	Resolved persistent COVID		Ongoing persistent COVID	
				≥12 to <52 weeks<br>No. (%)	≥52 weeks<br>No. (%)	≥12 to <52 weeks<br>No. (%)	≥52 weeks<br>No. (%)
No	92,527 (89.2)	96,644 (88.8)	5,798 (83.0)	1,768 (82.9)	695 (86.3)	1,662 (71.6)	2,070 (71.4)
Yes	11,260 (10.9)	12,187 (11.2)	1,189 (17.0)	365 (17.1)	110 (13.7)	659 (28.4)	831 (28.7)

PHQ-2 score is calculated by assigning scores of 0, 1, 2, and 3, to the response categories for two questions. *p<0·0001.

References

1. Office for National Statistics. Population Estimates for the UK, England and Wales, Scotland and Northern Ireland: mid-2020 2021. Available at: https://www.ons.gov.uk/peoplepopulationandcommunity/birthsdeathsandmarriages/deaths/articles/comparisonsofallcausemortalitybetweeneuropeancountriesandregions/januarytojune2020. Accessed 3 August 2022.
2. Office for National Statistics. Employee earnings in the UK: 2019 2019. Available at: https://www.ons.gov.uk/releases/employeeearningsintheuk2019. Accessed 3 August 2022.
3. Elliott P, Bodinier B, Eales O, Wang H, Haw D, Elliott J, et al. Rapid increase in Omicron infections in England during December 2021: REACT-1 study. Science. 2022;375(6587):1406-11.
4. UK Health Security Agency. SARS-CoV-2 variants of concern and variants under investigation in England. 2021. Available at: https://assets.publishing.service.gov.uk/government/uploads/system/uploads/attachment_data/file/1025827/Technical_Briefing_25.pdf. Accessed 18 March 2022.
5. Elliott P, Haw D, Wang H, Eales O, Walters CE, Ainslie KEC, et al. Exponential growth, high prevalence of SARS-CoV-2, and vaccine effectiveness associated with the Delta variant. Science. 2021;374(6574):eabl9551.
