# Supplementary material for: Long-term health impacts of COVID-19 among 242,712 adults in England
Source: Nat Commun. 2023 Oct 24;14:6588. doi: 10.1038/s41467-023-41879-2 (PMC10598213; doi:10.1038/s41467-023-41879-2)
Supplement: Supplementary file 3 — Reporting Summary [file 41467_2023_41879_MOESM3_ESM.pdf]

nature portfolio

Corresponding author(s): Professor Paul Elliott

Last updated by author(s): Sep 4, 2023

Reporting Summary

Nature Portfolio wishes to improve the reproducibility of the work that we publish. This form provides structure for consistency and transparency in reporting. For further information on Nature Portfolio policies, see our Editorial Policies and the Editorial Policy Checklist.

Statistics

For all statistical analyses, confirm that the following items are present in the figure legend, table legend, main text, or Methods section.

n/a	Confirmed	
☐	☒	The exact sample size (*n*) for each experimental group/condition, given as a discrete number and unit of measurement
☒	☐	A statement on whether measurements were taken from distinct samples or whether the same sample was measured repeatedly
☐	☒	The statistical test(s) used AND whether they are one- or two-sided<br>*Only common tests should be described solely by name; describe more complex techniques in the Methods section.*
☐	☒	A description of all covariates tested
☒	☐	A description of any assumptions or corrections, such as tests of normality and adjustment for multiple comparisons
☐	☒	A full description of the statistical parameters including central tendency (e.g. means) or other basic estimates (e.g. regression coefficient) AND variation (e.g. standard deviation) or associated estimates of uncertainty (e.g. confidence intervals)
☐	☒	For null hypothesis testing, the test statistic (e.g. *F*, *t*, *r*) with confidence intervals, effect sizes, degrees of freedom and *P* value noted<br>*Give P values as exact values whenever suitable.*
☒	☐	For Bayesian analysis, information on the choice of priors and Markov chain Monte Carlo settings
☒	☐	For hierarchical and complex designs, identification of the appropriate level for tests and full reporting of outcomes
☒	☐	Estimates of effect sizes (e.g. Cohen's *d*, Pearson's *r*), indicating how they were calculated

Our web collection on statistics for biologists contains articles on many of the points above.

Software and code

Policy information about availability of computer code

Data collection	Not applicable
Data analysis	STATA version 15·0

For manuscripts utilizing custom algorithms or software that are central to the research but not yet described in published literature, software must be made available to editors and reviewers. We strongly encourage code deposition in a community repository (e.g. GitHub). See the Nature Portfolio guidelines for submitting code & software for further information.

Data

Policy information about availability of data

All manuscripts must include a data availability statement. This statement should provide the following information, where applicable:

- Accession codes, unique identifiers, or web links for publicly available datasets
- A description of any restrictions on data availability
- For clinical datasets or third party data, please ensure that the statement adheres to our policy

The datasets generated or analysed, or both, during the current study are not publicly available because of governance restrictions and the identifiable nature of the data. Requests for access to raw data from the study should be addressed to the corresponding authors and will be answered within 12 weeks.The third party data provided by NHS Digital cannot be made available due to the conditions of the Data Sharing Agreement between Imperial College London and NHS England.

Data from NHS England can be requested directly, see https://digital.nhs.uk/services/data-access-request-service-dars.

Research involving human participants, their data, or biological material

Policy information about studies with human participants or human data. See also policy information about sex, gender (identity/presentation), and sexual orientation and race, ethnicity and racism.

Reporting on sex and gender	Data on participant sex was obtained from their NHS registration held by NHS England (the list of people registered with a General Practitioner). Analysis is presented by male/female categories.
Reporting on race, ethnicity, or other socially relevant groupings	Self-reported ethnicity was collected from participants. The coding of the ethnicity variable is described and it is used as a covariate in the analysis.
Population characteristics	Detailed breakdown of the characteristics of the study population are provided in the results tables and supplementary files. Age breakdown of study population - 18 to 24 (2.8%), 25 to 34 (6.6%), 35 to 44 (11.5%), 45 to 54 (17.4), 55 to 64 (25.8%), 65 to 74 (24.9%) and 75+ (11.0%). Sex at birth breakdown of study population – male (41.6%) and female (58.4%). COVID status breakdown of study population – No COVID (45.0%), Asymptomatic or resolved short COVID <4 weeks (48.2%), Resolved short COVID ≥4 to <12 weeks (3.1%), Resolved persistent COVID ≥12 weeks (1.3%), Ongoing persistent COVID ≥12 weeks (2.4%).
Recruitment	We obtained a sample of 800,000 adults aged ≥18 years using as a sample frame REACT-1 and REACT-2 participants who had consented to both re-contact and data linkage (n= 2,494,309). Personalised invitations were sent via email for one round of data collection between 1 August–1 December 2022. Participants registered via an online portal. Those registered completed an online questionnaire. Our questionnaire response rate was 34·6%. Our participants were more likely to be female, older, of white ethnicity and from the least deprived areas compared with the general adult population. These issues might cause selection bias in our study; however, we did not observe substantial differences between those invited and those who participated in the study on the measured sociodemographic characteristics. A further limitation is that we do not present estimates for population prevalence of persistent symptoms. To do so would require weighting but production of weights is far from straightforward given the composition of our sample. The probability of being in the sample was dependent upon the composition of the base population, varying response rates by sociodemographic group and across REACT 1 and REACT 2 rounds. We also oversampled participants who tested positive for SARS-CoV-2 and who reported persistent symptoms. Producing weights that take account of all these factors would involve making extensive assumptions which would likely introduce unknown biases.
Ethics oversight	Ethical approval for the study was obtained from South-Central Berkshire B Research Ethics Committee (IRAS IDs: 298404, 259978, 283787 and 298724).

Note that full information on the approval of the study protocol must also be provided in the manuscript.

Field-specific reporting

Please select the one below that is the best fit for your research. If you are not sure, read the appropriate sections before making your selection.

☐ Life sciences ☒ Behavioural & social sciences ☐ Ecological, evolutionary & environmental sciences

For a reference copy of the document with all sections, see nature.com/documents/nr-reporting-summary-flat.pdf

Behavioural & social sciences study design

All studies must disclose on these points even when the disclosure is negative.

Study description	A cross-sectional quantitative survey to investigate long-term physical and mental health impacts of COVID-19 on the adult England population. Follow up study of participants from the REACT programme.
Research sample	Participants from 19 rounds of the REACT-1 study and 6 rounds of the REACT-2 study who provided consent for re-contact and linkage to their administrative health data. Our participants were more likely to be female, older, of white ethnicity and from the least deprived areas compared with the general adult population and the sample frame.<br><br>Age breakdown of general adult population in England, UK - 18 to 24 (10.9%), 25 to 34 (17.3%), 35 to 44 (16.1%), 45 to 54 (17.5), 55 to 64 (15.0%), 65 to 74 (12.6%) and 75+ (10.5%). Sex at birth breakdown of of general adult population in England, UK – male (48.9%) and female (51.1%). Age breakdown of REACT-1 and -2 sample frame - 18 to 24 (5.7%), 25 to 34 (12.2%), 35 to 44 (15.3%), 45 to 54 (18.4), 55 to 64 (21.4%), 65 to 74 (18.4%) and 75+ (8.5%). Sex at birth breakdown of REACT-1 and -2 sample frame – male (44.9%) and female (55.1%). Age breakdown of study population - 18 to 24 (2.8%), 25 to 34 (6.6%), 35 to 44 (11.5%), 45 to 54 (17.4), 55 to 64 (25.8%), 65 to 74 (24.9%) and 75+ (11.0%). Sex at birth breakdown of study population – male (41.6%) and female (58.4%).
Sampling strategy	In this study, we aimed for a sample size of at least 160,000. Assuming a 20·0% response rate, we obtained a sample of 800,000 adults aged ≥18 years using as a sample frame REACT-1 and REACT-2 participants who had consented to both re-contact and data linkage (n= 2,494,309) To increase our sample of individuals with persistent symptoms of COVID-19 we first invited all individuals in the following subgroups:

	1. Individuals from REACT-1 or REACT-2 with a previous history of self-reported test confirmed or suspected COVID-19 who reported persistent symptoms of ≥12 weeks (n=52,501)<br>2. Individuals from REACT-1 who tested positive for SARS-CoV-2 as part of the study (n=13,482)<br>3. Individuals from REACT-2 who tested positive for SARS-CoV-2 IgG as part of the study and had not been vaccinated at the time (n=85,757)<br><br>To achieve the 800,000-participant size, a random sample (n=648,260) of all remaining adults not meeting the above criteria was selected.
Data collection	Data were collected by participants completing an online (or phone if requested) survey. No-one was present besides the participants and the researcher.<br><br>Linkage to administrative health data was undertaken for all participants who provided consent (described in the NHS Data Release Register, https://digital.nhs.uk/services/data-access-request-service-dars/data-uses-register, Reference number: DARS-NIC-431352-G7F1M-v0.4). Researchers were not blinded to hypothesis.
Timing	Survey data were collected between August and December 2022; administrative health data was obtained in August 2022.
Data exclusions	People who did not provide consent for recontact and administrative data linkage.
Non-participation	The overall response rate is 34·6%
Randomization	Randomisation not applicable to a cross-sectional study design.

Reporting for specific materials, systems and methods

We require information from authors about some types of materials, experimental systems and methods used in many studies. Here, indicate whether each material, system or method listed is relevant to your study. If you are not sure if a list item applies to your research, read the appropriate section before selecting a response.

Materials & experimental systems

n/a	Involved in the study	
☒	☐	Antibodies
☒	☐	Eukaryotic cell lines
☒	☐	Palaeontology and archaeology
☒	☐	Animals and other organisms
☒	☐	Clinical data
☒	☐	Dual use research of concern
☒	☐	Plants

Methods

n/a	Involved in the study	
☒	☐	ChIP-seq
☒	☐	Flow cytometry
☒	☐	MRI-based neuroimaging
